# Chronic lymphocytic infiltration with pontine perivascular enhancement responsive to steroids (CLIPPERS) and its association with Epstein‐Barr Virus (EBV)-related lymphomatoid granulomatosis: a case report

**DOI:** 10.1186/s12883-021-02110-1

**Published:** 2021-02-18

**Authors:** Yew Li Dang, Hong Kuan Kok, Penelope A. McKelvie, Matthew Ligtermoet, Laura Maddy, David A. Burrows, Douglas E. Crompton

**Affiliations:** 1 Department of Neurology, Northern Health, 185 Cooper Street, Epping, Victoria 3076 Australia; 2 Department of Radiology, Northern Health, Epping, Victoria 3076 Australia; 3 School of Medicine, Faculty of Health, Deakin University, Waurn Ponds, Victoria 3216 Australia; 4 Department of Anatomical Pathology, St Vincent’s Hospital, Fitzroy, Victoria 3065 Australia; 5 Department of Neurology, Austin Health, Heidelberg, Victoria 3084 Australia; 6 Department of Haematology, Northern Health, Epping, Victoria 3076 Australia; 7 Department of Medicine, University of Melbourne, Northern Health, Epping, Victoria 3076 Australia

**Keywords:** Chronic lymphocytic infiltration with pontine perivascular enhancement responsive to steroids, CLIPPERS, Lymphoma, Lymphomatoid granulomatosis, LYG, Epstein-Barr Virus, EBV

## Abstract

**Background:**

Chronic lymphocytic infiltration with pontine perivascular enhancement responsive to steroids (CLIPPERS) is a neuro-inflammatory syndrome first described in 2010. It has a relationship with lymphoproliferative disorders that has not been fully elucidated. This case represents an unusual progression of CLIPPERS to Epstein-Barr Virus (EBV)-related lymphomatoid granulomatosis (LYG). The exact connection between CLIPPERS and LYG remains poorly understood.

**Case presentation:**

We present a case of a 75-year-old man who was diagnosed with CLIPPERS with initial response to immunosuppression but later progressed to EBV-related LYG. EBV polymerase chain reaction (PCR) was detected in his cerebrospinal fluid (CSF), and repeat imaging revealed findings that were uncharacteristic for CLIPPERS; thereby prompting a brain biopsy which led to a diagnosis of EBV-related LYG. This case highlights the following learning points: 1) CLIPPERS cases are often part of a spectrum of lymphomatous disease, 2) CLIPPERS can be associated with EBV-related lymphoproliferative disorders such as LYG, and 3) EBV detection in CSF should prompt earlier consideration for brain biopsy in patients.

**Conclusions:**

Our case highlights the difficulty in distinguishing CLIPPERS from other steroid-responsive conditions such as neoplastic and granulomatous diseases. Given the association of CLIPPERS with EBV-related LYG as demonstrated in this case, we recommend testing for EBV in CSF for all patients with suspected CLIPPERS. An early referral for brain biopsy and treatment with rituximab should be considered for patients with suspected CLIPPERS who test positive for EBV in their CSF.

## Background

Chronic lymphocytic infiltration with pontine perivascular enhancement responsive to steroids (CLIPPERS) is an inflammatory central nervous system disorder first described in 2010 with a predilection for pontine involvement [1]. Since its description, increased awareness of this condition has led to more cases being diagnosed.

It remains unclear whether CLIPPERS represents a discrete disease entity or a spectrum of clinical conditions sharing a common neuroimmunological basis. We describe a case of a patient who initially presented with clinical and imaging features of CLIPPERS but later progressed to a diagnosis of Epstein-Barr virus (EBV) related-lymphomatoid granulomatosis (LYG). The exact association between CLIPPERS and LYG remains poorly understood.

## Case presentation

A 75-year-old man presented with a 3-week history of progressive vertigo, ataxia, dysarthria, dysphagia and hiccups; on the background of a 3-month history of fatigue, anorexia and 8 kilograms of unintentional weight loss. He had no significant past medical history and there was no history of taking immunosuppressive agents.

On examination, his Glasgow Coma Scale (GCS) was 15. There was significant truncal ataxia, bulbar dysarthria and direction-changing gaze-evoked horizontal nystagmus. Power was normal in the limbs, but there was severe ataxia and brisk deep tendon reflexes in both upper limbs. Heel–shin coordination was preserved, and lower limb reflexes and plantar responses were normal. He had an Expanded Disability Status Scale (EDSS) score of 6.5.

**Fig. 1:**
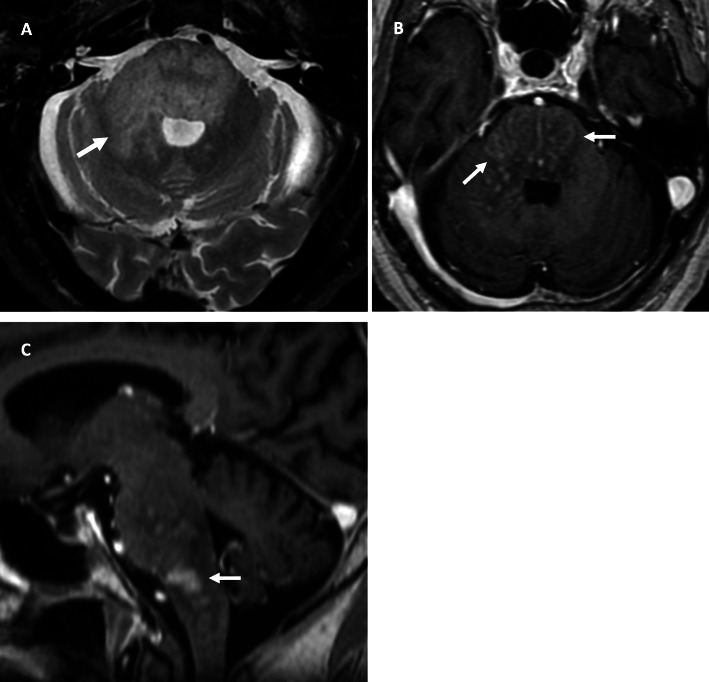
MRI brain at initial presentation. **a** Axial T2-weighted image of the brainstem shows diffuse signal change within the pons and right middle cerebellar peduncle (arrow). **b** Axial T1-weighted post-contrast image showing punctate and linear areas of enhancement (arrows) in the pons and right cerebellar peduncle. **c** Sagittal T1-weighted post-contrast image shows patchy enhancement in the pons and pontomedullary junction

Routine bloods were unremarkable. An infective screen including a tuberculosis interferon gamma release assay, and viral (hepatitis A/B/C, herpes simplex), schistosomal and strongyloides serology were all negative. Testing for human immunodeficiency virus which causes an immunocompromised state returned negative. Vasculitic, autoimmune and antineuronal antibody panels were also negative. Computed tomography (CT) of the brain was normal. A chest, abdomen and pelvis CT scan did not demonstrate any evidence of systemic malignancy. Cerebrospinal fluid (CSF) examination revealed elevated protein 0.76 g/L (0.15–0.40 g/L), glucose 4.6 mmol/L (2.5–4.5 mmol/L), 0 polymorphonuclear cells, 11 mononuclear cells, and 0 erythrocytes. Cytopathology revealed increased lymphocytes with no evidence of atypia. Flow cytometry was not possible due to low cell recovery. There were no bacteria seen; CSF culture was negative and *Mycobacterium tuberculosis* polymerase chain reaction (PCR) was negative. Matched oligoclonal immunoglobulin G (IgG) bands were detected in the CSF and serum. Magnetic resonance imaging (MRI) of the brain revealed diffuse signal change within the pons, cerebellar peduncles and pontomedullary junction with some mass effect, and characteristic punctate and linear enhancement typical for CLIPPERS (Fig. 1).

**Fig. 2:**
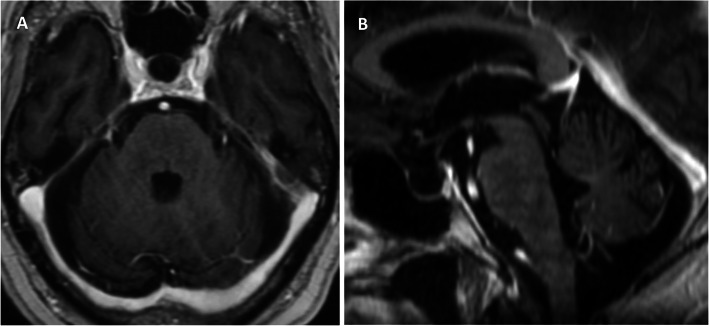
Follow-up MRI brain 4 months post commencing treatment. **a** Axial and **b** sagittal T1-weighted post-contrast images showing normal appearance of the pons, cerebellar peduncles and pontomedullary junction with no residual enhancement

Treatment with high-dose intravenous methylprednisolone (1 g/day) for five days, followed by a tapered dose of oral prednisolone (down to 20 mg/day) and an introduction of oral mycophenolate mofetil (500 mg BD), resulted in marked clinical improvement including restoration of normal mobility. His EDSS score improved to 1. A follow-up MRI brain revealed corresponding radiological resolution (Fig. 2). No atypical imaging features as previously described by Taieb et al. in their series [2] were evident on the baseline presentation and follow-up MRI brain studies. A diagnosis of CLIPPERS was made in view of the clinical presentation, radiological findings and exquisite response to steroid treatment.

Nine months later, our patient presented with a GCS of 10 (E3 V2 M5) and dysphasia after a 1-week history of lethargy. On examination, he had bilateral sixth nerve palsies and hyperreflexia in both upper limbs. His EDSS score worsened to 7. A lumbar puncture revealed elevated protein 0.57 g/L (0.15–0.40 g/L), normal glucose, 6 mononuclear cells, 0 polymorphs and 66 erythrocytes. No blasts or malignant cells were present. Flow cytometry was not performed due to low cell counts. CSF herpes simplex and enterovirus PCR were negative. John Cunningham virus serology was also negative. Notably, EBV PCR was positive in the CSF, with negative plasma EBV PCR. Oligoclonal IgG bands were detected in both CSF and serum; however, there were additional bands detected only in the CSF which were consistent with intrathecal synthesis.

**Fig. 3:**
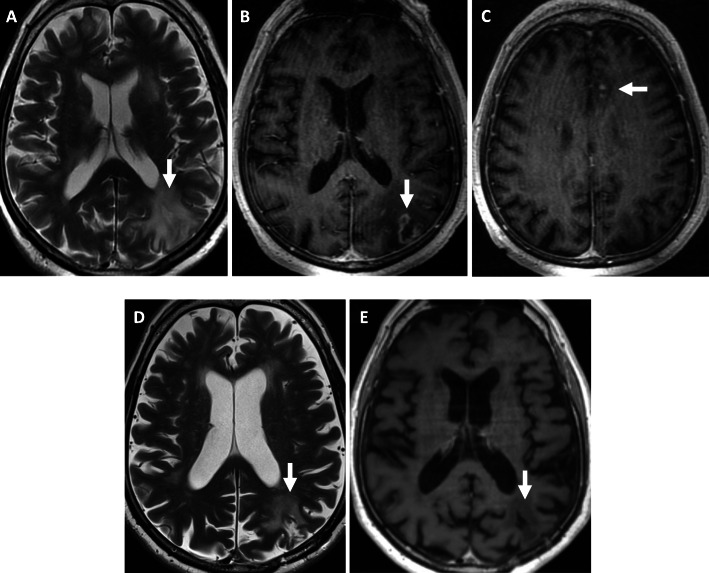
**a** Axial T2-weighted and **b** axial T1-weighted post-contrast images show a new rim-enhancing mass with vasogenic oedema in the left parietal lobe (arrow) and **c** patchy cortical enhancement in the left cingulate gyrus (arrow). Post-treatment MRI brain **d** axial T2-weighted and **e** T1-weighted post-contrast images show decrease in vasogenic oedema and reduced enhancement in the left parietal lobe compatible with treatment response

MRI of the brain revealed new peripherally enhancing lesions in the left frontal and parietal lobes (Fig. 3a-c). In addition, there were confluent T2 hyperintense changes in the bilateral periventricular white matter. Importantly, no new lesions were demonstrated in the pons or infratentorial compartment. These imaging findings are atypical for the progression of CLIPPERS and thus prompted a brain biopsy.

**Fig. 4:**
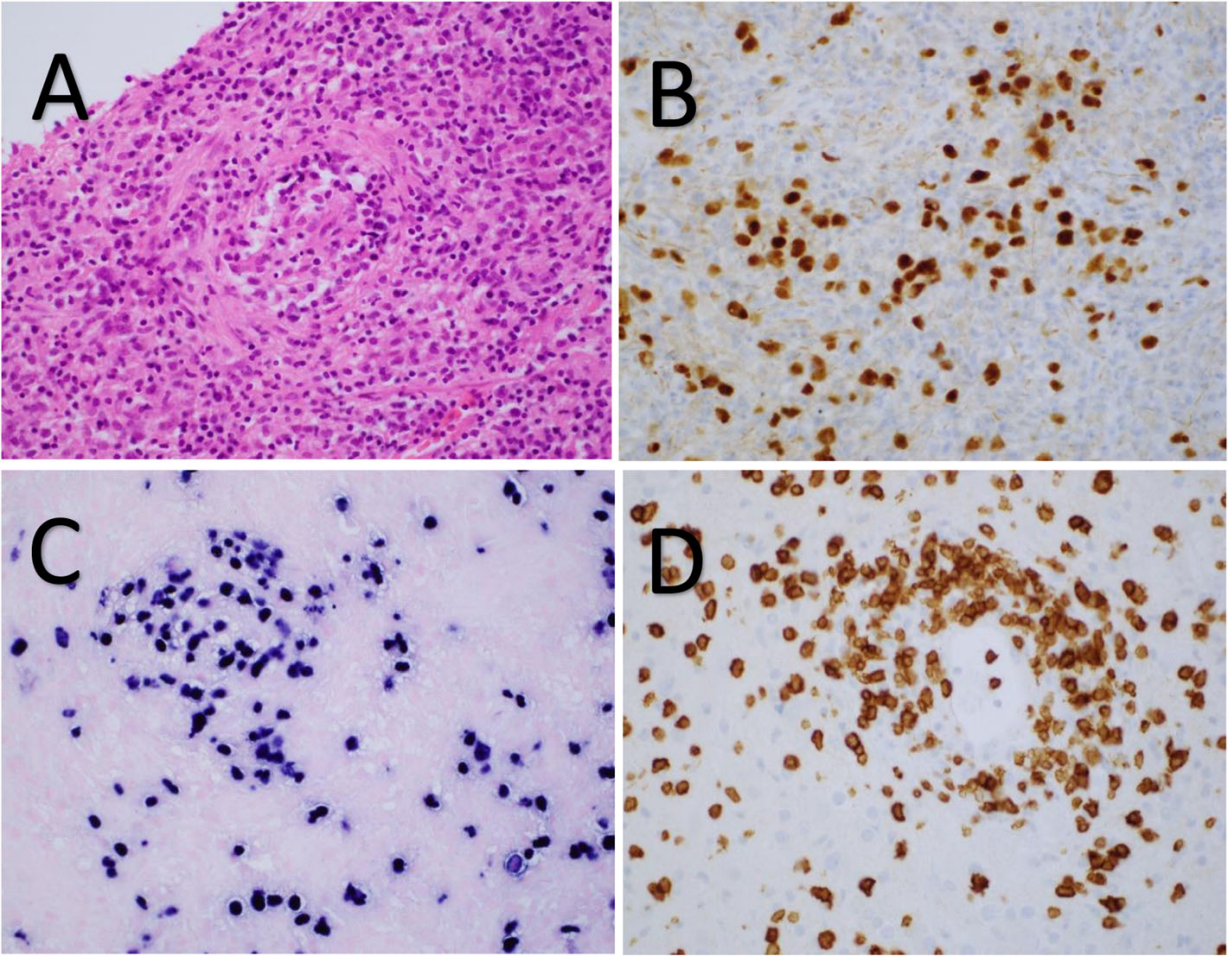
**a** High power magnification of angiocentric infiltrate of abnormal lymphoid cells and histiocytes in the brain, Haematoxylin and eosin. **b** High power magnification showing numbers of large B-cells with PAX5 immunohistochemistry. **c** Numbers of EBV+ large B-cells with Epstein-Barr Encoding Region In Situ Hybridisation (EBER ISH) on high power magnification. **d** Numerous small T-cells in the angiocentric infiltrate with CD3 immunohistochemistry

Biopsy of the left parietal lesion showed an angiocentric infiltrate of epithelioid histiocytes, lymphocytes and large atypical lymphoid cells with large areas of necrosis. The large atypical lymphoid cells were B-cells (CD20+, PAX5+, Epstein-Barr Encoding Region In Situ Hybridisation +) and the lymphocytes were predominantly small T-cells (CD3+) (Fig. 4a-d). The features were those of lymphomatoid granulomatosis grade III.

There was no evidence of any skin or pulmonary involvement of LYG. Our patient was enrolled in a treatment trial for lymphoma, and has since commenced treatment with ibrutinib, rituximab and EBV-specific cytotoxic T-cells (EBV-CTLs). He is tolerating his treatment well and has made significant clinical improvement. His GCS improved to 15 and he had an EDSS score of 2. His dysphasia has resolved and is fully independent with mobility. Similarly, his repeat MRI Brain six months post treatment also demonstrated a decrease in vasogenic oedema and reduced enhancement in the left parietal lobe compatible with treatment response (Fig. 3d-e).

## Discussion and conclusions

LYG is a rare angiocentric and angiodestructive B-cell lymphoproliferative disorder characterised histologically by a predominance of reactive T-cells and fewer neoplastic EBV-positive B-cells [3]. LYG range from an indolent process with no B-cell atypia (grade I) to an aggressive B-cell lymphoma (grade III) [3].

The exact connection between CLIPPERS and LYG is poorly understood. Possibilities include:

1. CLIPPERS representing an early phase of LYG. Some of these cases progress due to T-cell dysfunction inherent in CLIPPERS, resulting in reduced T-cell immunosurveillance [4].
2. CLIPPERS reflecting a host immune response against a primary central nervous system lymphoma, which could then remit spontaneously or with steroid therapy. Transformation to LYG happens when the host immune response is suppressed [5].
3. LYG represents an EBV-associated lymphoproliferative disorder which is usually associated with an underlying immunodeficiency [6]. Therefore, immunosuppression used in the treatment of CLIPPERS may contribute to the pathogenesis of LYG.

The association of EBV with LYG suggests a transformative role of EBV in LYG pathogenesis [6]. LYG is hypothesised to result from a defective immune surveillance of EBV [7]. In two published cases of CLIPPERS to LYG, both patients had EBV-positive cells in the second brain biopsy four months and three months after the first brain biopsies that were negative for EBV [4, 8]. However, one of these patients had received high dose IV methylprednisolone prior to the first biopsy [8], which is known to partly treat high grade B-cell lymphoma of the CNS. The other patient [4] had cerebellum biopsied first and then a necrotic pontine lesion biopsied second. The short time interval between two biopsies suggests that prior treatment or sampling may have obscured underlying LYG. One of these patients had CLIPPERS red flags on the initial episode with the “brain on fire” changes on MRI in the corpus callosum [4] and the other only showed atypical CLIPPERS clinical signs of peripheral nerve facial palsy on the second episode [8]. Of the three patients reported by Taieb et al., with non-CLIPPERS with LYG [2], their cases 3 and 4 had non-CLIPPERS MRI findings in the first episode, whereas their case 10 had probable CLIPPERS until developing relapse with large pontine mass on 80 mg /day of prednisolone and LYG on brain biopsy at four months. Interestingly, Taieb et al’s case 4 had suffered an EBV-related macrophage activation syndrome one year prior to presentation with CLIPPERS [2]. The transformative role of EBV has also been highlighted in a recent case report which reflects latent EBV infection’s role in converting CLIPPERS to EBV-positive diffuse large B cell lymphoma especially in the setting of chronic immunosuppression [9].

In our case, our patient did not have a brain biopsy at initial presentation, nor EBV testing on the initial CSF sample. However, during his second presentation, EBV was detected by PCR of the CSF and by Epstein-Barr Encoding Region in situ hybridization in the brain biopsy. but was undetectable in plasma. The presence of EBV in CSF or brain biopsy samples should prompt a search for alternative diagnoses such as EBV-related lymphoproliferative diseases. This is important given the risk of transformation to lymphomatoid granulomatosis in EBV positive patients [6], especially in the setting of immunosuppression as treatment for CLIPPERS. We recommend testing for EBV in CSF for patients with suspected CLIPPERS; and in those patients who test positive for EBV in their CSF, an early referral for brain biopsy should be considered.

Given the rarity of CLIPPERS, there is a lack of randomised controlled treatment trials. The evidence for the use of various steroid regimens and steroid-sparing agents remain anecdotal. Rituximab has proven efficacy in maintaining remission in CLIPPERS [10], and in the treatment and prevention of EBV-related lymphoproliferative disorders [11]. Rituximab should therefore be considered as a steroid-sparing agent for the management of patients with CLIPPERS and EBV positivity at the outset.

Although various clinical, radiological and pathological characteristics have been proposed to aid the diagnosis of CLIPPERS [12], distinguishing CLIPPERS from other steroid-responsive conditions such as neoplastic and granulomatous diseases remains challenging [13]. Patients with CLIPPERS associated with lymphoma have a worse prognosis compared to those without lymphoma [14]. Therefore, early consideration of CLIPPERS associated lymphoproliferative disorders is important. Given the association of CLIPPERS with EBV-related LYG, as demonstrated in this case, we recommend testing for EBV in CSF for all patients with suspected CLIPPERS. An early referral for brain biopsy and treatment with rituximab should be considered for patients with suspected CLIPPERS who test positive for EBV in their CSF.

## Data Availability

The datasets used and/or analysed during the current study are available from the corresponding author upon request.

## Abbreviations

CLIPPERS: Chronic lymphocytic infiltration with pontine perivascular enhancement responsive to steroids.
CSF: Cerebrospinal fluid
CT: Computed tomography
EBV: Epstein-Barr Virus
EDSS: Expanded Disability Severity Scale
EBER: Epstein-Barr Encoding Region
GCS: Glasgow Coma Scale
IgG: Immunoglobulin G
ISH: In situ hybridisation
LYG: Lymphomatoid granulomatosis
MRI: Magnetic resonance imaging
PCR: Polymerase chain reaction
